# Impact of mixed biofilm formation with environmental microorganisms on *E. coli* O157:H7 survival against sanitization

**DOI:** 10.1038/s41538-020-00076-x

**Published:** 2020-10-14

**Authors:** Sapna Chitlapilly Dass, Joseph M. Bosilevac, Maggie Weinroth, Christian G. Elowsky, You Zhou, Angela Anandappa, Rong Wang

**Affiliations:** 1 Department of Animal Science, Texas A&M University, College Station, TX 77845 USA; 2 U. S. Department of Agriculture, Roman L. Hruska U.S. Meat Animal Research Center, Clay Center, Lincoln, NE 68933-0166 USA; 3 Department of Agronomy and Horticulture, University of Nebraska – Lincoln, Lincoln, NE 68588 USA; 4 Center for Biotechnology, University of Nebraska – Lincoln, Lincoln, NE 68588 USA; 5 Alliance for Advanced Sanitation, Department of Food Science, University of Nebraska–Lincoln, Food Innovation Campus, Nebraska Lincoln, 68588 USA; 6 Present Address: Agricultural Research Service, Roman L. Hruska U.S. Meat Animal Research Center, Clay Center, Nebraska Lincoln, 68933-0166 USA

**Keywords:** Industrial microbiology, Biofilms

## Abstract

Biofilm formation by foodborne pathogens is a serious threat to food safety and public health. Meat processing plants may harbor various microorganisms and occasional foodborne pathogens; thus, the environmental microbial community might impact pathogen survival via mixed biofilm formation. We collected floor drain samples from two beef plants with different *E. coli* O157:H7 prevalence history and investigated the effects of the environmental microorganisms on pathogen sanitizer tolerance. The results showed that biofilm forming ability and bacterial species composition varied considerably based on the plants and drain locations. *E. coli* O157:H7 cells obtained significantly higher sanitizer tolerance in mixed biofilms by samples from the plant with recurrent *E. coli* O157:H7 prevalence than those mixed with samples from the other plant. The mixed biofilm that best protected *E. coli* O157:H7 also had the highest species diversity. The percentages of the species were altered significantly after sanitization, suggesting that the community composition affects the role and tolerance level of each individual species. Therefore, the unique environmental microbial community, their ability to form biofilms on contact surfaces and the interspecies interactions all play roles in *E. coli* O157:H7 persistence by either enhancing or reducing pathogen survival within the biofilm community.

## Introduction

Many foodborne pathogens such as *Escherichia coli* O157:H7, *Salmonella enterica*, and *Listeria monocytogenes* can form biofilms. This presents a serious food safety concern for meat processing plants because viable pathogens in detached biofilms from contact surfaces can lead to cross-contamination. The environmental biofilms are most often composed of multispecies microorganisms and such microbial community is shaped by the environment in which it grows. Importantly, available studies have shown that mixed biofilm formation can enhance sanitizer tolerance of foodborne pathogens^1,2^.

Floor drains at meat processing plants have been recognized as an important niche that may harbor foodborne pathogens such as *L. monocytogenes*^3,4^. Due to the poor accessibility and difficulty for regular maintenance of hygiene status, floor drains may contain a wide variety of environmental microorganisms, within which a high percentage of the biofilm-forming population can be isolated^4^. The composition of bacterial species in floor drains is most likely dynamic and influenced by the drain location, processing activity, and the cleaning/sanitization procedures applied. Furthermore, biofilm contamination of drainage pipes or water resources can be amplified by the ability of bacteria to swim upstream, using the so-called “rheotaxis” mechanism^5^. The rinsing water from animal carcasses, processing equipment, and other environmental surfaces that may contain foodborne pathogens can accumulate in the drains, thus, the variety of microorganisms found in floor drains is a good representation of the bacterial species present in the processing environment. Mixed biofilm formation by the environmental microorganisms and foodborne pathogens can potentially provide an ecological niche for the pathogens to better colonize and obtain a higher survival capability against routine sanitization procedures. As a result, pathogen prevalence and the risk of product contamination in meat plants may be increased.

Notably, available results have shown that the different genus types of the environmental microorganisms can significantly affect the synergistic or antagonistic interactions among the resident microflora, and subsequently can either promote or inhibit the growth and colonization of specific pathogens in the mixed biofilm matrix. For instance, a recent study observed synergistic interactions among bacteria isolated from household dishwashers. *Acinetobacter junii* and *Pseudomonas aeruginosa* appeared to be the major contributors to enhanced mixed biofilm formation that could further promote biofilm establishment by the opportunistic fungal pathogen *Exophiala dermatitidis*^6^. Fox et al.^7^ also observed genera variations in bacterial populations collected from floor drains with or without the colonization by *Listeria* species, showing that genera *Prevotella* and *Janthinobacterium* were closely associated with the *Listeria*-negative samples whereas *Enterococcus* and *Rhodococcus* were in higher abundance in *Listeria*-positive samples. This study further revealed that biofilm formation by *Listeria monocytogenes* was inhibited by the presence of *Janthinobacterium* but conversely, *Enterococcus gallinarum* significantly enhanced *Listeria* biofilm development^7^.

The above findings led us to hypothesize that the interspecies interactions among the different resident microflora in meat processing facilities would either enhance or inhibit pathogen survival and persistence, which could in turn affect the prevalence rate of the pathogens such as *E. coli* O157:H7, the most commonly identified Shiga-toxin producing *E. coli* associated with foodborne outbreaks. In the beef industry, some processing plants may experience intermittent or persistent higher rates of *E. coli* O157:H7 than other plants with no directly identifiable cause. Between the two plants selected for the present study, Plant A has had a historically higher prevalence rate of *E. coli* O157:H7 compared to Plant B. During the sample collection period, beef trim positive rate of *E. coli* O157:H7 at Plant B was lower than 0.1%, which is considered as the normal sporadic level. Meanwhile, Plant A exhibited an overall higher positive rate of 0.5–1.0%, mostly due to a recurring number of “high event period” (HEP) contamination incidence during which a 3–10% positive rate was observed; thus, increasing its average trim *E. coli* O157:H7-positive rate. Interestingly, available studies suggested that in-house biofilm formation by *E. coli* O157:H7 might contribute to HEP contamination^8–10^.

Therefore, we collected floor drain samples from these two plants then analyzed and compared the impact of environmental microorganisms on *E. coli* O157:H7 survival in mixed biofilms derived from these drain samples. The environmental samples were collected from floor drains located in coolers (where carcasses are stored for sorting before fabrication) and hotboxes (where carcasses are spray-chilled and stored immediately after exiting the harvest floor) from both facilities. We phenotypically and genetically characterized and compared the bacteria from the drain samples for their biofilm-forming ability under meat processing conditions. Furthermore, an *E. coli* O157:H7 strain was inoculated into each of the drain communities to form mixed biofilms, followed by treatment with common quaternary ammonium chloride (QAC) sanitizer. The post-sanitization survival of the *E. coli* O157:H7 strain in the different drain communities was compared to assess the potential impact of the environmental microorganisms on *E. coli* O157:H7 sanitizer tolerance. The pre- and post-sanitization biofilm samples were also analyzed by 16S rRNA gene amplicon sequencing to determine if the composition of bacterial species in the samples was related to *E. coli* O157:H7 survival.

## Results

### Biofilm formation by floor drain microorganisms

**Fig. 1:**
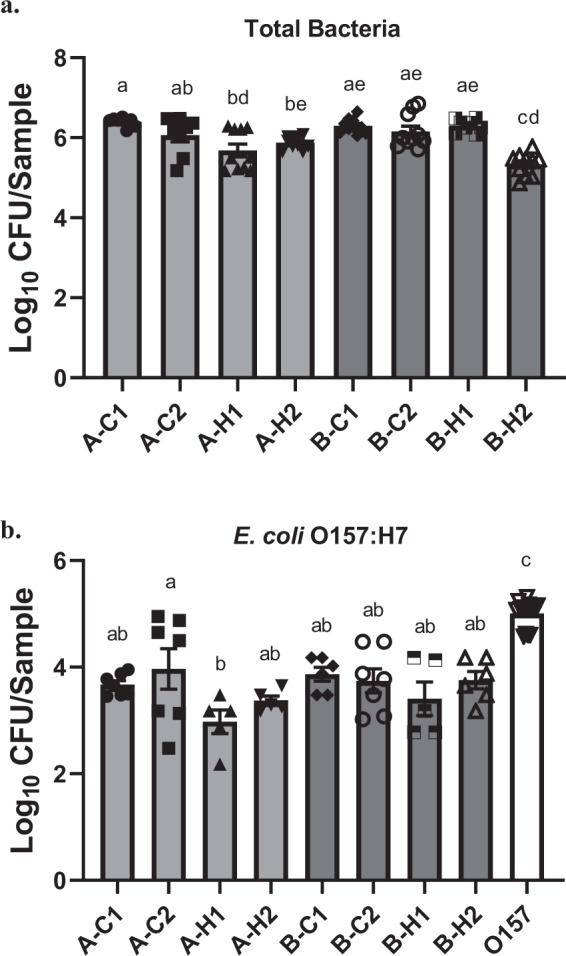
Viable total bacteria or *E. coli* O157:H7 cells in biofilms treated with sterile water. **a** Viable total bacteria in mixed biofilms formed by floor drain samples. **b** Viable *E. coli* O157:H7 cells in mixed biofilms or single-strain biofilm. Viable total bacteria and *E. coli* O157:H7 cells in sterile water-treated biofilms were harvested and quantified on TSA and O157 Chromagar plates, respectively. Data are shown as log_10_ CFU/chip. Error bars represent mean ± standard error of the mean. Statistical analysis was performed using ANOVA followed by post Turkey’s multiple comparisons test. *P*-values lower than 0.05 were considered statistically significant. Values labeled with the same letter are not statistically different.

All drain samples containing environmental microorganisms developed a significant biofilm matrix on stainless steel (SS) surface at 7 °C, and the biofilm-forming ability was sample-dependent (Fig. 1a). Overall, the mean of total biofilm cells recovered from the SS surface ranged from 5.3 to 6.4 log_10_ CFU/chip. The highest biofilm matrix was observed in cooler sample A-C1 from Plant A, and cooler sample B-C1 and hotbox sample B-H1 from Plant B. The hotbox sample B-H2 from Plant B formed the least biofilm mass (5.3 log_10_ CFU/chip) among the eight samples. Notably, all four samples collected from cooler drains at both processing plants developed high biofilm mass (>6.0 log_10_ CFU/chip).

### Biofilm development by *E. coli* O157:H7 strain in mixed biofilms

The amount of *E. coli* O157:H7 cells recovered in mixed biofilms co-cultured with each drain sample ranged from 2.9 to 4.0 log_10_ CFU/chip. Among the eight mixed biofilms containing the *E. coli* O157:H7 strain, the only significant difference in the amount of *E. coli* O157:H7 cells recovered was observed between samples A-C2 (cooler drain sample from Plant A) and A-H1 (hotbox drain sample from Plant A). The cell density of the single-strain *E. coli* O157:H7 biofilm on SS surface reached ~5.0 log_10_ CFU/chip, which was significantly higher than those recovered from the mixed biofilms containing the *E. coli* O157:H7 strain (Fig. 1b).

### Confocal laser scanning microscopy

**Fig. 2:**
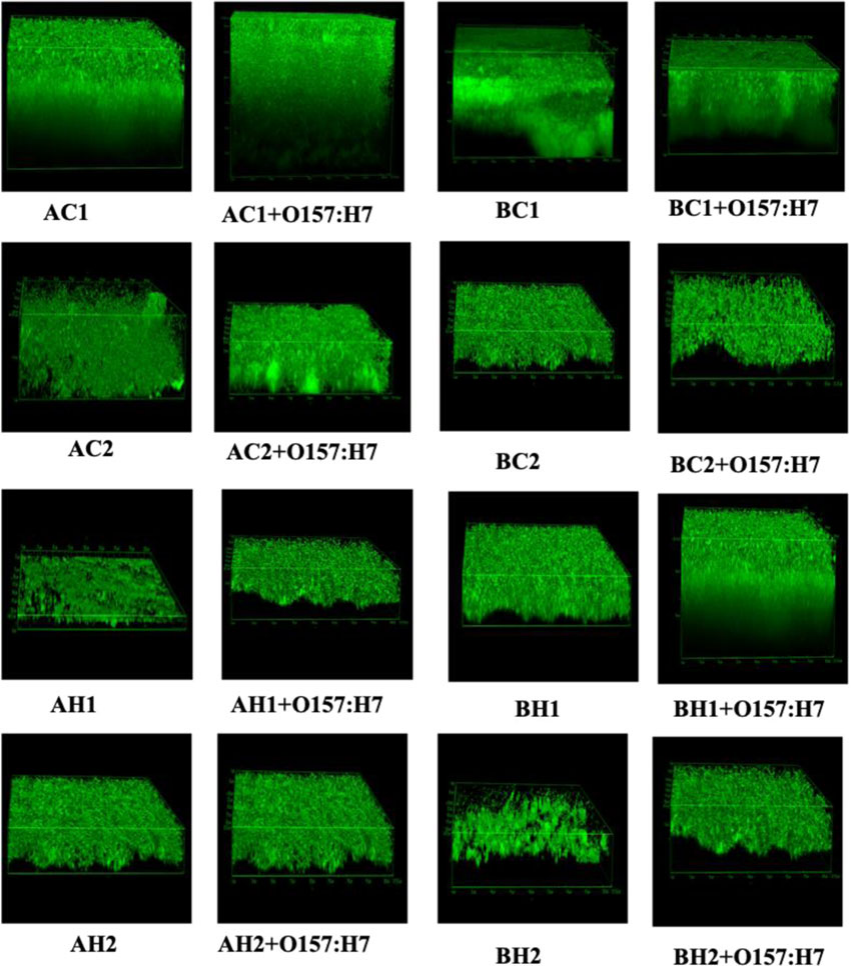
CLSM 3D images of mixed biofilms on a stainless steel surface. Mixed biofilms developed by the drain microorganisms, with or without the addition of the *E. coli* O157:H7 strain, were stained with FM 1–43 dye and the structural organization of the biofilms was analyzed with CLSM and the software package. Calibration *XY*: 0.21 μm, *Z*: 0.75 μm. Resolution: 1024 × 1024 × 29.

The CLSM images of the FM 1–43 dye stained biofilms developed by the drain microorganisms on stainless steel chips, with and without the addition of the *E. coli* O157:H7 strain, are shown in Fig. 2. The 3D images project the thickness of the biofilms that ranged from 20.19 µm to 157.55 µm (Supplementary Table 1). The biofilm architecture and bacterial density correlated positively to the amounts of bacterial cells measured by colony enumeration on agar plates. Notably, a significant difference in the thickness of the biofilms before and after the incorporation of *E. coli* O157:H7 was observed. The thickness of the biofilm mass increased after the addition of *E. coli* O157:H7 to the mixed biofilm community. Plant A drain sample A-C1 with higher bacterial diversity formed biofilm with significantly greater biomass, was sanitizer tolerant, and also protected *E. coli* O157:H7. In contrast, Plant B sample B-H2, that had the lowest species diversity, was susceptible to sanitization and provided the least protection to the *E. coli* O157:H7, formed very thin biofilm as evident in Fig. 2. We attribute the biomass difference to bacterial aggregation within the mixed biofilms and their ability to adhere to stainless steel, the commonly used material in food-processing facilities.

### Sanitizer tolerance of biofilms by drain microorganisms

**Fig. 3:**
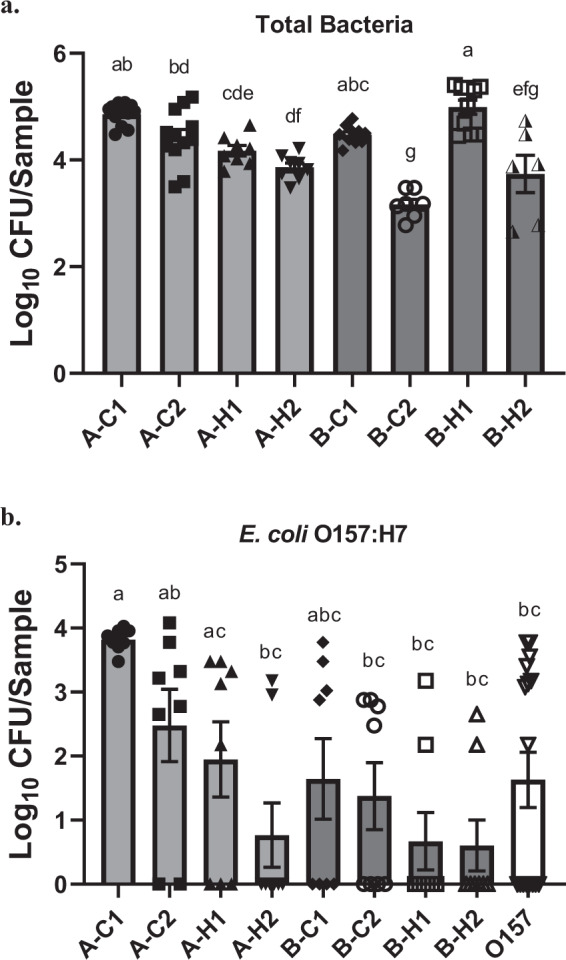
Viable total bacteria or *E. coli* O157:H7 cells in biofilms treated with 300 ppm QAC. **a** Viable total bacteria in mixed biofilms by floor drain samples. **b** Viable *E. coli* O157:H7 cells in mixed biofilms or single-strain biofilm. Viable total bacteria and *E*. *coli* O157:H7 cells in QAC-treated biofilms were harvested and quantified on TSA and O157 Chromagar plates, respectively. Data are shown as log_10_ CFU/chip. Error bars represent mean ± standard error of the mean. Statistical analysis was performed using ANOVA followed by post Turkey’s multiple comparisons test. *P*-values lower than 0.05 were considered statistically significant. Values labeled with the same letter are not statistically different.

After 300 ppm QAC treatment, the amounts of viable total bacteria cells in biofilms by each individual drain sample were reduced by 1.3–3.0 log_10_ CFU/chip. Maximum log reductions (>2.0 log) with QAC treatment was observed in biofilms formed by hotbox sample A-H2 from Plant A (2.0 log_10_ CFU/chip), and cooler sample B-C2 (3.0 log_10_ CFU/chip) from Plant B that had the lowest amount of surviving bacteria (Fig. 3a). For all other samples, the treatment reduced biofilm cell density by 1.3–1.8 log_10_ CFU/chip (Table 1).

**Table 1:** Locations/names of the floor drain samples and bacterial log reductions in mixed biofilms after 300 ppm QAC treatment.

Plant	A				B				O157 only
Drain location	Cooler 1	Cooler 2	Hotbox 1	Hotbox 2	Cooler 1	Cooler 2	Hotbox 1	Hotbox 2	
Sample name	A-C1	A-C2	A-H1	A-H2	B-C1	B-C2	B-H1	B-H2	
Biofilml log reduction^a^	1.5 (0.1)	1.6 (0.2)	1.5 (0.2)	2.0 (0.1)	1.8 (0.1)	3.0 (0.2)	1.3 (0.1)	1.6 (0.4)	
O157 log reduction^b^	−0.2 (0.1)	1.5 (0.7)	1.0 (0.6)	2.6 (0.5)	2.2 (0.6)	2.4 (0.6)	2.7 (0.5)	3.2 (0.4)	3.4 (0.4)

^a^Log reduction (log_10_ CFU/chip ± SD) of total bacterial counts after 300 ppm QAC treatment in mixed biofilms by environmental microorganisms collected from floor drains.

^b^Log reduction (log_10_ CFU/chip ± SD) of viable *E. coli* O157:H7 cells after 300 ppm QAC treatment in mixed biofilms by environmental microorganisms collected from floor drains, or in *E. coli* O157:H7 single-strain biofilm.

### Survival of *E. coli* O157:H7 cells in single-strain and mixed biofilms

Survival of the *E. coli* O157:H7 strain in mixed biofilms after QAC treatment was variable and highly dependent upon the drain sample with which the strain was co-cultured. The least amount of viable *E. coli* O157:H7 cells after the treatment was observed when the strain was mixed with hotbox drain sample A-H2 (0.8 log_10_ CFU/chip), B-H1 (0.7 log_10_ CFU/chip) and B-H2 (0.6 log_10_ CFU/chip). Conversely, the maximum amount of viable *E. coli* O157:H7 cells that survived the treatment reached 3.8, 2.5, and 2.0 log_10_ CFU/chip when the strain was co-cultured with cooler drain samples A-C1 and A-C2, and hotbox drain sample A-H1, respectively, all collected from Plant A (Fig. 3b). *E. coli* O157:H7 cells surviving in mixed biofilm O157/A-C1 was significantly higher (*P* < 0.05) than those recovered from other mixed biofilms except for samples O157/A-C2, O157/A-H1, and O157/B-C1. Importantly, compared to the *E. coli* O157:H7 cells surviving in single-strain biofilm, the only significantly higher amount of viable *E. coli* O157:H7 cells after QAC treatment was observed in the mixed biofilm co-cultured with cooler drain sample A-C1 (Fig. 3b).

### Bacterial communities in the drain-associated biofilms

**Fig. 4:**
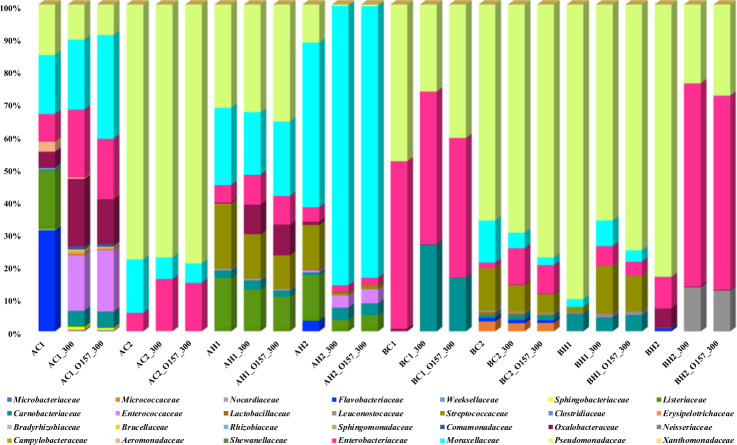
Impact of QAC treatment on species relative abundance changes of the mixed biofilms. For each drain biofilm sample, one pre-treatment sample (A-C1–B-H2), one post-treatment sample without *E. coli* O157:H7 (A-C1_300–B-H2_300), and one post-treatment sample with the addition of the *E. coli* O157:H7 strain (A-C1_O157_300–B-H2_O157_300) were analyzed with 16S rRNA gene amplicon sequencing to demonstrate the changes in the relative abundance of microorganisms in mixed biofilms before and after QAC treatment.

For each floor drain sample, one pre-treatment biofilm sample, one post-treatment biofilm sample with *E. coli* O157:H7, and one post-treatment biofilms without the addition of *E. coli* O157:H7 strain, were sequenced. Therefore, a total of 24 drain-associated biofilm samples were analyzed to assess the bacterial communities before and after the QAC treatment (Fig. 4). A total of 3,572,808 partial 16S rRNA gene sequences were obtained after demultiplexing from the 24 biofilm samples. Sequences were de-replicated into unique amplicon sequence variants (ASV; traditionally referred to as OTUs) and a list of representative sequences was created with 251 features.

The relative abundance of sequences of pre-QAC-treated biofilms by bacteria derived from each individual drain sample was assigned to 28 different families, of which *Pseudomonadacea, Moraxellaceae*, and *Enterobacteriaceae* were present in all drain biofilms sequenced. Sample A-C1, a potent biofilm former (6.4 log_10_ CFU/chip) and the strongest protector of *E. coli* O157:H7, contained the highest diversity of bacterial species among the eight samples and included the dominant taxa of *Flavobacteriacea*, *Moraxellacea*, *Listeriacea*, *Pseudomonacea*, *Enterobacteriaceae*, *Weeksellacea*, *Sphingobacteriaceae*, and *Aeromonadaceae*. The dominant taxa in sample A-C2 were *Enterobacteriaceae*, *Pseudomonacea*, and *Moraxellacea*. For the two samples collected from hotbox drains at Plant A, the dominant taxa in sample A-H1 included *Listeriaceae*, *Streptococcaceae*, *Enterobacteriaceae*, *Moraxellaceae*, and *Pseudomonadaceae*, and in sample A-H2 were *Listeriaceae*, *Carnobacteriaceae*, *Streptococcaceae*, *Enterobacteriaceae*, *Moraxellaceae*, and *Pseudomonadaceae*.

In Plant B, between the two strong biofilm formers that both reached 6.3 log_10_ CFU/chip, hotbox sample B-H1 had higher diverse taxa compared to cooler sample B-C1. The dominant taxa in sample B-H1 included *Carnobacteriaceae*, *Pseudomonadaceae*, *Moraxellaceae*, and *Streptococcaceae*. In sample B-C1 the dominant taxa were *Enterobacteriaceae* and *Pseudomonadaceae*. The weakest biofilm former, hotbox sample B-H2, had the dominant taxa of *Enterobacteriaceae, Moraxellaceae*, and *Pseudomonadaceae*.

### Effect of QAC treatment on bacterial diversity

The impact of QAC treatment on species relative abundance changes of each sample is illustrated in Fig. 4. With the incorporation of *E. coli* O157:H7 and QAC treatment, the relative abundance of certain taxa in sample A-C1 increased while some other dominant taxa decreased. The dominant taxa *Flavobacteriacea* and *Listeriaceae* were reduced after treatment with QAC and the addition of *E. coli* O157:H7; meanwhile, there was an increase in the relative abundance of *Carnobacteriaceae, Micrococcaceae*, and *Enterococcaceae* that were present in lower abundance prior to QAC treatment. Relative abundance increase was also observed in *Brucellaceae* and *Sphingobacteriaceae*, the two families shared by the three strong *E. coli* O157:H7 protectors A-C1, A-C2, and A-H1. Relative abundance reduction of *Pseudomonadaceae*, *Flavobacteriacea*, *Listeriaceae*, *Streptococcaceae*, and *Oxalobacteraceae* was observed in sample A-H2 after QAC treatment.

The relative abundance was not significantly impacted after the *E. coli* O157:H7 strain addition and QAC treatment in sample A-C2 and B-C2, except minor increases of *Carnobacteriaceae* and *Enterobacteriaceae* in sample A-C2 and *Pseudomonadaceae* in sample B-C2. In sample A-H1 and B-H1, there was a significant increase in the relative abundance of *Oxalobacteraeae* and *Enterobacteriaceae*, respectively.

### Bacterial community composition and *E. coli* O157:H7 tolerance

To further understand the role of the species diversity and community composition in *E. coli* O157:H7 tolerance, the drain samples were classified into two groups based on the log reductions of the pathogen: the protectors (samples A-C1, A-C2, and A-H1) and the non-protectors (A-H2, B-C1, B-C2, B-H1, and B-H2). The three strong *E. coli* O157:H7 protectors from Plant A had shared families *Weeksellaceae, Sphingibacteriaceae*, and *Brucellaceae*. Notably, the strongest protector A-C1, which had the highest species diversity among the eight pre-treated samples, contained 7 unique families (Supplementary Table 2). Four weak *E. coli* O157:H7 protectors (A-H2, B-C2, B-H1, B-H2) all had *Lactobacillaceae* and *Leuconostocaseae*, members of the lactic acid bacteria (LAB) family.

**Fig. 5:**
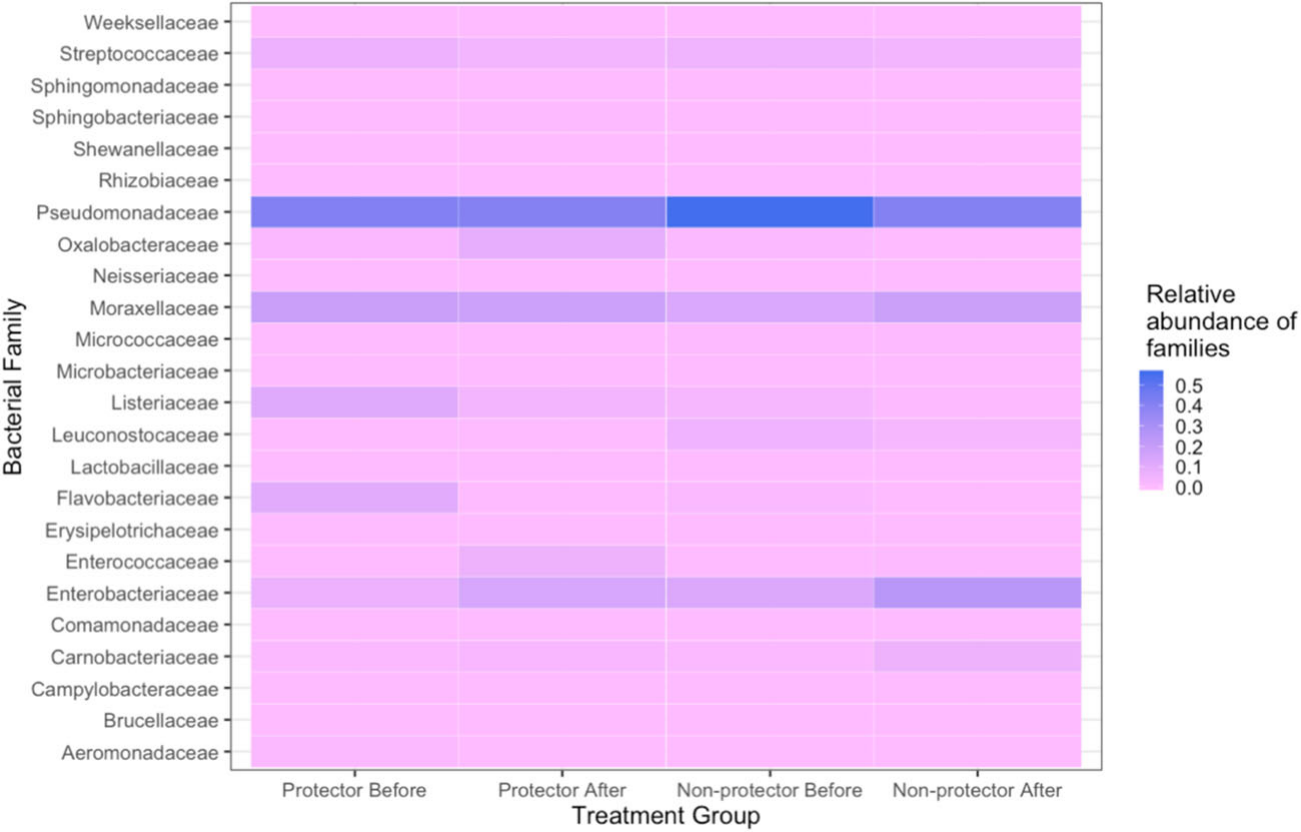
Proportion of relative abundance of the bacterial species in the O157 protector and non-protector groups. Heatmap is created by normalizing the OUT table counts, that are converted to the proportions of relative abundance of the bacterial families present in the microbiome grouped by *E. coli* O157:H7 protector status before and after QAC treatment.

The proportion of relative abundance of the bacterial families in each group before and after QAC treatment is shown in Fig. 5. After treatment, the proportions of *Enterococcaceae* and *Oxalobacteraceae* were considerably increased in the protectors. *Enterococcaceae* was also the most increased family in the strongest protector A-C1. Conversely, the relative abundance of *Listeriaceae* and *Flavobacteriaceae* were decreased in the protector group. In the non-protector group, the relative abundance of the lactic acid bacteria family members *Lactobacillaceae* and *Leuconostocaceae*, which were previously reported to inhibit *E. coli* O157:H7 biofilm development^11–13^, were decreased.

**Fig. 6:**
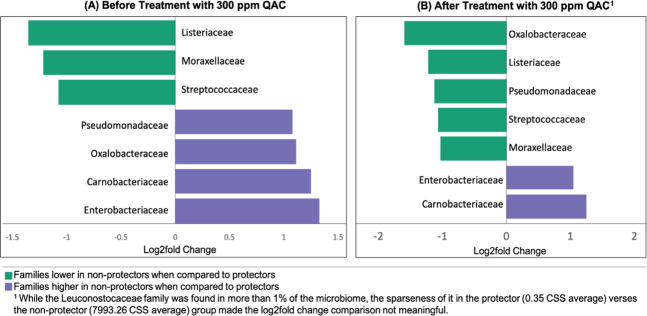
Log_2_ fold differences in the relative abundance of bacterial families between the O157 protector and non-protector groups. Log_2_ fold change differences between the sample groups at the family level were calculated on log_2_ transformed CSS normalized counts. Only bacterial families present in more than 1% of the total microbiome and differing by more than 1.0 log between the sample groups before (**a**) and after (**b**) QAC treatment are presented.

The log_2_ fold difference in the relative abundance of bacterial family composition between the protector and the non-protector groups before and after QAC treatment is shown in Fig. 6. The majority of the families with significant log_2_ fold difference between the two groups remained the same after sanitization except for *Pseudomonadaceae* and *Oxalobacteraceae*. These two families were overall in higher abundance in pre-treated non-protectors, however, QAC treatment reduced the relative abundance of the two families in the non-protector samples.

## Discussion

Meat processing plants harbor a variety of microorganisms as well as occasional foodborne pathogens, and a large portion of such a microbial community can persist in the environment as multispecies biofilms. Survival of pathogens such as *E. coli* O157:H7 in these niches as part of the mixed biofilms depends on the interspecies interactions within the microbial community. Previous studies have shown that multispecies biofilms can enhance the survival of *E. coli* O157:H7 that had become an established member of the community^1,2^. Recent findings^9,14^ have further suggested biofilm formation in commercial meat plants may be a source of product contamination. Since floor drains collect all rinsing water and liquid wastes in the plants, the microbial communities in the floor drains represent the microecological niches that encompass the various microorganisms in the processing plant environment.

We collected floor drain samples from two meat processing plants with different prevalence rates of *E. coli* O157:H7 to study the impact of mixed biofilms by environmental microorganisms on pathogen survival and persistence. Our results are consistent with previous findings^1,2^ that after sanitization the *E. coli* O157:H7 cells in its single-strain biofilm were reduced more than those in mixed biofilms. More importantly, a trend was observed that there were overall higher amounts of viable *E. coli* O157:H7 cells in QAC-treated biofilms mixed with Plant A samples than those with Plant B samples. Such protective effects on the pathogen were not directly associated with the overall tolerance level of the entire mixed biofilm community (Table 1), nor was it solely dependent upon the total biovolumes of the mixed biofilm matrix (Fig. 2).

Mixed biofilms are formed in the environment through synergistic interactions within the microbial community through mutual adaptive responses to their long-term coexistence^15^. The individual microbial species in the community are also recruited based on their co-evolution in the shared niche^6,16^. We observed that the inoculated *E. coli* O157:H7 strain was able to establish itself in the mixed biofilms efficiently (Fig. 1b), however, the amount of *E. coli* O157:H7 cells in all mixed biofilms were significantly (*P* < 0.05) lower than that recovered from the *E. coli* O157:H7 single-strain biofilm. Given that these complex environmental samples contained high microbial diversity, the interspecies interactions would have a significant role in determining the levels of member species in the mixed biofilm. Such an observation is in agreement with previous studies that various bacteria, including non-pathogenic strains of *E. coli*^8^, have antagonistic effects on biofilm formation by *E. coli* O157:H7 in mixed cultures. This is likely due to the competition for limited nutritional resource and colonization space, or the production of toxic metabolites and inhibitory substances by the other bacterial strains in the mixture.

It has been known that species spatial organization within the mixed biofilm 3D structure can greatly affect bacterial survival since such 3D architecture may act as a physical barrier blocking sanitizer penetration. A recent study^17^ that investigated the influence of interspecies interactions on the spatial organization of member species in mixed biofilms indicated that the presence of *Microbacterium oxydans* as a key species, even though in low abundance, could act as a driving force to strongly increase the overall biovolume of a four-species mixed biofilm. More importantly, *M. oxydans* could impact the spatial organization of the mixed biofilm and stabilize the structure of the entire community, resulting in certain partner species located in the top layers of the mixture. As described above, unique species of sample A-C1 in low abundance, including the *Microbacteriaceae* family, may also have critical roles in species spatial organization of O157/A-C1 mixed biofilms and benefit partner strains including the *E. coli* O157:H7 cells from such arrangement, as the so-called “layered biofilm structure” could offer protection for species hidden underneath^14^. Future studies using fluorescent-tagged bacteria and time-lapse imaging are required to investigate the spatial distribution of the critical species as well as the location of *E. coli* O157:H7 cells inside the mixed biofilm structure.

In addition to biofilm architecture, bacterial species diversity, and their ability to adhere, compete, and form biofilms on common processing materials can impact pathogen tolerance^2,9^. *Enterococcaceae*, for instance, the most abundant family in the strongest protector A-C1 after sanitization, was also previously found closely associated with *Listeria*—colonized drain environment^6^. The relative abundance of *Flavobacteriaceae*, on the other hand, was decreased in the protector group. Interestingly, a previous multispecies biofilm study revealed that this family was abundant during early succession with a high growth rate, suggesting its ability to outcompete companion strains at the initial colonization stage^11^, which was consistent with our observations.

The complexity and variability of the interactions within the multispecies communities may increase with the number of species present in the mixture. This may lead to a more complex biofilm structure and a potentially more comprehensive mechanism against sanitization resulting from cooperation among the various species/strains in the community. Environmental species diversity reflects the various microhabitats found at the locations and the different selective pressure resulting from the sanitizing/cleaning routines. Different chemical agents were used between the two processing plants. The antimicrobial agents applied on beef carcasses can influence the bacterial community profile on the carcass surface^18^. While the carcasses enter the hotbox to chill, this process is accelerated through the intermittent application of automated spray chilling. Some processors use municipal water supply, while others incorporate an antimicrobial such as chlorine or peroxyacetic acid. The chemicals in the spray chill water may carry away carcass surface bacteria and concentrate them in floor drains where these various factors would further shape the community.

The high prevalence and recurrence of *E. coli* O157:H7 in Plant A environment could also be an adaptive response to long-term coexistence, the so-called “memory effect” or “history of co-habitation”. This phenomenon was previously reported by Madsen et al.^16^. who demonstrated that the synergistic interactions and bacterial social behaviors within mixed biofilms were related to their prior coexistence as the microbial species were recruited into the community based on their co-evolution in the shared niche^6,16^. The adaptable responses to bacterial recurrence could be the driving force for community recruitment of the species that were present frequently in the past and co-evolved in the local environment^16^. Therefore, pathogens removed during sanitation of the facility, when reintroduced into the same environment, could be effectively recruited back into the mixed biofilm community, leading to recurrent contamination of the environment. Thus, it is reasonable to speculate that the unique microbial diversity, the history of co-habitation, and the local selective pressure at Plant A might help recruit and protect *E. coli* O157:H7, increase its survival and persistence, leading to the higher *E. coli* O157:H7 prevalence at Plant A compared to Plant B.

Conversely, the lower prevalence history of *E. coli* O157:H7 in Plant B could also be related to its local microbial dynamics and interactions, as well as the unique environmental species diversity such as the presence of lactic acid bacteria. Previous reports suggested that lactic acid bacteria could competently compete with *E. coli* O157:H7, *Salmonella enterica* and *Listeria monocytogenes* due to the production of antimicrobial substances and free radicals, as well as the ability to co-aggregate with the pathogens in mixed biofilms^11–13^. Thus, a natural alternative for pathogen biofilm prevention has been suggested using lactic acid bacteria as probiotic strains to develop protective biofilms for the inhibition, exclusion and displacement of pathogenic biofilms. The contribution of the lactic acid bacteria members to the low *E. coli* O157:H7 survival in Plant B and their potential use as probiotic biofilm formers in the meat industry warrant further studies.

In summary, our results suggest that the environmental microorganisms in meat processing plants may affect the colonization and the subsequent sanitizer tolerance of *E. coli* O157:H7. The fact that Plant A had a history of recurrent *E. coli* O157:H7 prevalence may be associated with its history of co-habitation with the resident microbes, and the local higher species diversity that was better able to protect *E. coli* O157:H7 cells in mixed biofilms. Conversely, certain species at Plant B might be related to the low survival and prevalence of the pathogen via competition and inhibition. Future studies are required to further dissect the microbial communities derived from the floor drain samples and identify the specific species and mechanisms that either protect or inhibit the pathogen. Understanding how microorganisms function as a community to either cooperate for mutual benefit or to compete for survival will help in rationally designing niche-specific antimicrobial interventions in food-processing facilities.

## Methods

### Drain sample collection and characterization

Floor drain samples were collected from two meat processing plants (Table 1) using cellulose sponges (Speci-sponge; Nasco, Atkinson WI) each wetted with 10 mL buffered peptone water (BPW). Drains on opposing areas (e.g., north versus south) of hotbox and cooler were identified that were at least 30 m apart and connected to separate drainage lines. Hotbox drains were those in the portion of the beef processing plant where carcasses that immediately exit the harvest floor are spray-chilled to ~1 °C and stored for 24 to 48 h. Cooler drains were those in the portion of the beef processing plant where carcasses are graded, sorted, and stored at ~1 °C before fabrication. The time a carcass spends in the cooler can range from 1 to 72 h depending on carcass grade (e.g., prime, choice, select, or other) and the processor’s daily operations. Processing plant A had a combination of side-trap drains and standard industrial drains located at low points in the floor for the collection and removal of liquid (Supplementary Fig. 1A, B). Hotbox samples at plant A were collected from side-trap drains while the cooler samples were collected from industrial drains. Processing plant B had trench-type drains, where a 6 in (15 cm) wide and 6 in deep (15 cm) grate-covered trough crossed the floor. Trench drains channeled liquid to a drain and trap centrally located along the trough (Supplementary Fig. 1C). All samples collected at Plant B were from trench drains. When an appropriate drain was identified, the covering grate and any interior strainer or basket was removed. Then an area of ~500 cm^2^ was sampled to collect bacteria and biofilms. The underside of the grate, sides of the basket if present, and/or undersides of side-trap entry vents, as well as interior surfaces, were sampled by vigorous swabbing with the sponge. Halfway through the collection the sponge was turned over. Sponges were placed in a whirl-pak bag and transported back to the laboratory on wet ice in a beverage cooler. To ensure an adequate sample was collected from each drain, total Aerobic plate count (APC), Psychophilic bacteria (PB), and *Enterobacteriaceae* (EB) were enumerated using appropriate Petrifilm (3 M, St Paul, MN), incubated at 35 °C for APC and EB or 7 °C for PB. Samples were further confirmed to be free of *E. coli* O157:H7 using O157 Chromagar plates (DRG International Inc., Mountainside, NJ) as *E. coli* O157:H7 would form unique pink colonies on the O157 Chromagar plates that can be distinguished from other background microorganisms^19^.

### Culture conditions for drain samples and the *E. coli* O157:H7 strain

To simulate the fabrication environment at the meat plants and maintain the microbial composition of the floor drain samples, each sample was 50-fold inoculated into Lennox Broth (LB, Acumedia Manufacturers, Baltimore, MD) without salt (LB-NS) medium and incubated at 7 °C for 5 days with orbital shaking at 200 rpm, then aliquoted and stored at −20 °C in LB-NS medium with the addition of sterile glycerol to 15%.

The *E. coli* O157:H7 strain used in this study was isolated from naturally contaminated ground beef manufacturing trim^10^. The strain was stored at −70 °C in LB-NS medium containing 15% glycerol. Prior to use, the strain was streaked from the glycerol stock onto Tryptic Soy Agar (TSA) (Difco, Beckton Dickinson, Sparks, MD) plates and grown overnight at 37 °C. Bacterial broth culture at stationary phase was prepared by inoculating one single colony on TSA plate into LB-NS medium and grown overnight at 37 °C with orbital shaking at 200 rpm to reach a cell concentration of ~5 × 10^8^ cells/mL, then further diluted in fresh sterile LB-NS medium for each experiment^2,20^.

### Sanitizer

The quaternary ammonium chloride (QAC)-based sanitizer Vanquish^TM^ (Dawn Chemical Corp., Milwaukee, WI), a common commercial sanitizer product authorized by the USDA as category D2 for use in meat, poultry, and other food-processing plants, was applied in this study. This sanitizer contains an alkylbenzyldimethyl—ammonium chloride mixture as its active ingredients. A working solution of Vanquish ^TM^ (300 ppm) was prepared following the manufacturer’s instructions.

### Biofilm formation and sanitizer treatment

To investigate whether mixed biofilm formation with environmental microorganisms from the different floor drains would affect sanitizer tolerance of *E. coli* O157:H7 cells, biofilms by the drain microorganisms, with or without the addition of *E. coli* O157:H7 cells, and *E. coli* O157:H7 cells alone were developed on stainless steel (SS) chips and quantified by colony enumeration method on agar plates^2,21^. To do so, the glycerol stocks of the floor drain samples stored at −20 °C as described above were thawed, diluted 1000-fold, inoculated into sterile LB-NS medium then incubated at 7 °C for 5 days with orbital shaking at 200 rpm. On the fifth day, a 1.0 mL aliquot was removed from each sample, diluted in sterile LB-NS medium, and plated onto TSA and O157 Chromagar plates for colony enumeration after overnight incubation at 37 °C. The 5-day cultures plated on the Chromagar plates would also further confirm that no false-positive pink colonies were observed in any of the drain samples before the addition of the *E. coli* O157:H7 strain.

The remaining 5-day cultures were aliquoted into 50 mL centrifuge tubes, 15 mL per tube, then one sterile SS (18 × 18 × 2 mm) chip was placed in each of the tubes to be immersed in the culture as a platform on which biofilms were developed statically for another 5 days at 7 °C. To prepare mixed biofilm samples containing the *E. coli* O157:H7 strain, an overnight culture of the *E. coli* O157:H7 strain containing approximately 5 × 10^8^ CFU/mL cells was added into the 5-day drain cultures in the tubes at a 1:100 ratio and incubated with the SS chips for mixed biofilm development. In addition, single-strain biofilms formed by the *E. coli* O157:H7 strain alone were included as controls by incubating the SS chips with a 100-fold diluted overnight culture of the *E. coli* O157:H7 strain in LB-NS medium for 5 days at 7 °C.

At the end of the incubation period, each chip was rinsed with 10 mL sterile water, 5 mL each side, then immersed in sterile water (control) or 300 ppm QAC solution for one minute, which is the recommended minimal exposure time for QAC treatment. To neutralize the sanitizer activity, each chip was then transferred to a new 50 mL centrifuge tube containing 10 mL Dey/Engley broth (BBL, Difco, Sparks, MD) supplemented with 0.3% soytone and 0.25% sodium chloride, and 1gram sterile glass beads (425–600 microns; Sigma-Aldrich, St. Louis, MO). Biofilm cells were removed from the chip surface with 1 min sonication followed by 2 min vortexing at maximal speed, then 10-fold serially diluted in sterile Dey/Engley broth and plated onto TSA (biofilms by drain samples only) or O157 ChromAgar plates (biofilms by drain samples with the addition of the O157 strain) for colony enumeration after overnight incubation at 37 °C. The amount of *E. coli* O157:H7 cells in mixed biofilms, distinguished from background microorganisms by colony morphology on O157 ChromAgar plates as described above, were compared among the various mixed biofilm samples and the *E. coli* O157:H7 single-strain biofilm to determine whether background microflora collected from each drain environment would provide the pathogen protection against sanitization.

### DNA extraction and 16S rRNA gene amplicon-based sequencing

Biofilm cells were harvested from the SS surface as described above and centrifuged at 13,000 × *g* at 4 °C for 5 min. The supernatants were gently removed, and the cells were resuspended in phosphate-buffered saline (PBS) followed by a second round of centrifugation at 13,000 × *g* for 2 min. The obtained cell pellets were processed for DNA extraction and purification using the Power soil microbial DNA isolation kit (Mo Bio Laboratories, Inc., Carlsbad, CA) following the manufacturer’s protocol. DNA concentrations were quantified using Qubit dsDNA assay kit (double-stranded DNA-HS high sensitivity assay) and the fluorescence was measured on a Qubit fluorometer (Life Technologies Corporation, Carlsbad, CA).

To characterize bacterial diversity from the biofilm cell DNA, amplicon sequencing based on the variable region V4 of the 16S rRNA gene was performed. Primers used were 15F (5′-GTGCCAGCMGCCGCGGTAA-3′) and 806R (5′-GGACTACHVGGGTWTCTAAT3′), flanking the 515 and 806 regions. Barcodes were attached to the 806R primers. Library preparation and 2 × 250 bp paired-end sequencing was carried out using the Illumina® MiSeq® platform at NeoSeek^TM^ (Neogen, Lincoln, NE)^22^.

### Confocal laser scanning microscopy (CLSM)

SS chips with biofilms developed on surfaces as described above were transferred to six-well cell culture plates and gently washed with filter-sterilized molecular grade water and then stained with FM 1–43 dye (1:1000 in filter-sterilized water) (Life Technologies, Eugene, Oregon, USA) for 15 min in the dark. The samples were then washed with filter-sterilized water to remove the dye, stored in filter-sterilized water at 4 °C, and imaged on the same day. For imaging, each chip stained with FM 1–43 dye was covered with an 18 × 18 mm glass coverslip and imaged using Nikon MRD07602 model CLSM with an excitation of 488 nm and an emission of 500–550 nm. A ×60 lens CFI Plan Apochromat VC 60X WI (WI water immersion) was used for magnification. *Z*-stacks were acquired for each biofilm sample^23^. The structural organization of the biofilms was analyzed using the Comstat2 software package (http://www.comstat.dk)^24^. The 3D representations of the biofilm structures were generated using the 3D viewer plugin for the FIJI distribution of ImageJ (http://3dviewer.neurofly.de).

### Statistical analysis

The amount of biofilm cells was measured as log_10_ CFU/chip by calculation using colony-forming units (CFU) on the agar plates and their corresponding dilution factors. Analysis of variance and comparisons of the logarithmic biofilm cell counts with standard deviations and 95% confidence intervals were performed using GraphPad Prism software (GraphPad Software, La Jolla, CA). Logarithmic cell counts recovered from mixed biofilms by the various floor drain samples and the *E. coli* O157:H7 single-strain biofilm were analyzed and compared using a one-way analysis of variance (ANOVA) with a post-Tukey’s or Dunnett’s multiple comparisons test. Standard deviation (SD) of log reductions between pre- and post-sanitization samples of total bacterial counts or viable *E. coli* O157:H7 cells was calculated using the formula SD = sqrt (SD_pre_^2^/*n*_pre_ + SD_post_^2^/*n*_post_). *P*-values lower than 0.05 were considered statistically significant.

Analysis of 16S rRNA gene sequencing results was conducted using Quantitative Insights Into Microbial Ecology (QIIME2.0) (https://qiime2.org/). Paired-end sequences were de-multiplexed using MiSeq Control software prior to importing into QIIME. FastQC was used to check sample sequence quality. Based on the quality checks, forward reads were truncated to 240 bp and reverse reads were truncated to 200 bp. Taxonomy was assigned using the Greengenes database with the pre-trained classifier based on a 99% sequence identity.

The heatmap and log_2_ fold difference between the sample groups were generated from the constructed OTU tables collapsed to the family level using R v. 3.5.2. The heatmap was created by CSS normalizing the OTU table counts (via metagenomeSeq v. 1.24.1) which were then converted to proportions. The heatmap was generated using ggplot2 v. 3.1.1. Only bacterial families present in more than 1% of the total microbiome were considered in log_2_ fold difference comparisons. Log_2_ fold change differences between sample groups at the family level were calculated on log_2_ transformed CSS normalized counts using gtools’ (v. 3.8.1) fold change function.

## Supplementary information

Supplementary material

## Data Availability

The authors declare that the data supporting the findings of this study are presented within the manuscript and the supplementary information files. Additional data sources such as the volume snapshots for the 3D confocal laser microscopy and the fastq.qz files of the Illumina paired-end sequencing are also available from the corresponding author upon reasonable request.
