# Supplementary material for: Impact of mixed biofilm formation with environmental microorganisms on E. coli O157:H7 survival against sanitization
Source: NPJ Sci Food. 2020 Oct 14;4:16. doi: 10.1038/s41538-020-00076-x (PMC7560865; doi:10.1038/s41538-020-00076-x)
Supplement: Supplementary file 1 — Supplementary material [file 41538_2020_76_MOESM1_ESM.pdf]

Supplementary Table 1

Biofilm ID	X (µm)	Y (µm)	Z (µm)
AC1	212.12	212.12	69.75
AC1+ O157:H7	212.12	212.12	157.55
AC2	212.12	212.12	30
AC2+ O157:H7	212.12	212.12	49.25
AH1	212.12	212.12	34.60
AH1+ O157:H7	212.12	212.12	20.25
AH2	212.12	212.12	22.20
AH2+ O157:H7	212.12	212.12	55.47
BC1	212.12	212.12	89.75
BC1+ O157:H7	212.12	212.12	94.30
BC2	212.12	212.12	35.1
BC2+ O157:H7	212.12	212.12	42.6
BH1	212.12	212.12	73.65
BH1+ O157:H7	212.12	212.12	91.16
BH2	212.12	212.12	20.19
BH2+ O157:H7	212.12	212.12	24

Supplementary Table 2 (part 1)

Organism	AC1	AC1_300	AC1_O157_300	AC2	AC2_300	AC2_O157_300	AH1	AH1_300	AH1_O157_300	AH2	AH2_300	AH2_O157_300
Actinobacteria;c_Actinobacteria;o_ Actinomycetales;f_Microbacteriaceae	y	y	y									
Actinobacteria;c_Actinobacteria;o_ Actinomycetales;f_Micrococcaceae	y	y	y				y	y	y	y	y	y
Actinobacteria;c_Actinobacteria;o_ Actinomycetales;f_Nocardiaceae												
Bacteroidetes;c_Flavobacteriia;o_ Flavobacteriales;f_Flavobacteriaceae	y	y	y							y	y	
Bacteroidetes;c_Flavobacteriia;o_ Flavobacteriales;f_[Weeksellaceae]	y	y	y	y	y	y	y	y	y			
Bacteroidetes;c_Sphingobacteriia;o_ Sphingobacteriales;f_Sphingobacteriaceae	y	y	y	y	y	y	y	y	y			
Firmicutes;c_Bacilli;o_ Bacillales;f_Listeriaceae	y	y	y				y	y	y	y	y	y
Firmicutes;c_Bacilli;o_ Lactobacillales;f_Carnobacteriaceae	y	y	y				y	y	y	y	y	y
Firmicutes;c_Bacilli;o_ Lactobacillales;f_Enterococcaceae	y	y	y				y	y	y	y	y	y
Firmicutes;c_Bacilli;o_ Lactobacillales;f_Lactobacillaceae										y	y	y
Firmicutes;c_Bacilli;o_ Lactobacillales;f_Leuconostocaceae										y	y	y
Firmicutes;c_Bacilli;o_ Lactobacillales;f_Streptococcaceae	y	y	y				y	y	y	y	y	y
Firmicutes;c_Clostridia;o_ Clostridiales;f_Clostridiaceae												
Firmicutes;c_Erysipelotrichi;o_ Erysipelotrichales;f_Erysipelotrichaceae	y	y	y									
Proteobacteria;c_Alphaproteobacteria;o_ Rhizobiales;f_Bradyrhizobiaceae												
Proteobacteria;c_Alphaproteobacteria;o_ Rhizobiales;f_Brucellaceae	y	y	y	y	y	y	y	y	y			
Proteobacteria;c_Alphaproteobacteria;o_ Rhizobiales;f_Rhizobiaceae	y	y	y									
Proteobacteria;c_Alphaproteobacteria;o_ Sphingomonadales;f_Sphingomonadaceae	y	y	y									
Proteobacteria;c_Betaproteobacteria;o_ Burkholderiales;f_Comamonadaceae	y	y	y									
Proteobacteria;c_Betaproteobacteria;o_ Burkholderiales;f_Oxalobacteraceae	y	y	y				y	y	y	y	y	y
Proteobacteria;c_Betaproteobacteria;o_ Neisseriales;f_Neisseriaceae							y	y	y			
Proteobacteria;c_Epsilonproteobacteria;o_ Campylobacterales;f_Campylobacteraceae												
Proteobacteria;c_Gammaproteobacteria;o_ Aeromonadales;f_Aeromonadaceae	v	v	v									
Proteobacteria;c_Gammaproteobacteria;o_ Alteromonadales;f_Shewanellaceae	v	v	v									
Proteobacteria;c_Gammaproteobacteria;o_ Enterobacteriales;f_Enterobacteriaceae	y	y	y	y	y	y	y	y	y	y	y	y
Proteobacteria;c_Gammaproteobacteria;o_ Pseudomonadales;f_Moraxellaceae	y	y	y	y	y	y	y	y	y	y	y	y
Proteobacteria;c_Gammaproteobacteria;o_ Pseudomonadales;f_Pseudomonadaceae	y	y	y	y	y	y	y	y	y	y	y	y

Unique Famalies
Shared families in samples that were strong O157:H7 protectors
Shared families in samples that were weak O157:H7 protectors

Supplementary Table 2 (part 2)

Organism	BC1	BC1_300	BC1_O157_300	BC2	BC2_300	BC2_O157_300	BH1	BH1_300	BH1_O157_300	BH2	BH2_300	BH2_O157_300
Actinobacteria;c_Actinobacteria;o_ Actinomycetales;f_Microbacteriaceae												
Actinobacteria;c_Actinobacteria;o_ Actinomycetales;f_Micrococcaceae				y	y	y	y	y	y			
Actinobacteria;c_Actinobacteria;o_ Actinomycetales;f_Nocardiaceae	y	y	y									
Bacteroidetes;c_Flavobacteriia;o_ Flavobacteriales;f_Flavobacteriaceae	y	y		y	y	y	y	y	y	y	y	y
Bacteroidetes;c_Flavobacteriia;o_ Flavobacteriales;f_[Weeksellaceae]												
Bacteroidetes;c_Sphingobacteriia;o_ Sphingobacteriales;f_Sphingobacteriaceae												
Firmicutes;c_Bacilli;o_ Bacillales;f_Listeriaceae												
Firmicutes;c_Bacilli;o_ Lactobacillales;f_Carnobacteriaceae		y	y	y	y	y	y	y	y			
Firmicutes;c_Bacilli;o_ Lactobacillales;f_Enterococcaceae							y	y	y			
Firmicutes;c_Bacilli;o_ Lactobacillales;f_Lactobacillaceae				y	y	y	y	y	y	y	y	y
Firmicutes;c_Bacilli;o_ Lactobacillales;f_Leuconostocaceae				y	y	y	y	y	y	y	y	y
Firmicutes;c_Bacilli;o_ Lactobacillales;f_Streptococcaceae				y	y	y	y	y	y		y	y
Firmicutes;c_Clostridia;o_ Clostridiales;f_Clostridiaceae										y	y	
Firmicutes;c_Erysipelotrichi;o_ Erysipelotrichales;f_Erysipelotrichaceae												
Proteobacteria;c_Alphaproteobacteria;o_ Rhizobiales;f_Bradyrhizobiaceae										y		
Proteobacteria;c_Alphaproteobacteria;o_ Rhizobiales;f_Brucellaceae												
Proteobacteria;c_Alphaproteobacteria;o_ Rhizobiales;f_Rhizobiaceae												
Proteobacteria;c_Alphaproteobacteria;o_ Sphingomonadales;f_Sphingomonadaceae												
Proteobacteria;c_Betaproteobacteria;o_ Burkholderiales;f_Comamonadaceae												
Proteobacteria;c_Betaproteobacteria;o_ Burkholderiales;f_Oxalobacteraceae	y	y	y		y	y				y	y	y
Proteobacteria;c_Betaproteobacteria;o_ Neisseriales;f_Neisseriaceae												
Proteobacteria;c_Epsilonproteobacteria;o_ Campylobacterales;f_Campylobacteraceae	y	y	y									
Proteobacteria;c_Gammaproteobacteria;o_ Aeromonadales;f_Aeromonadaceae												
Proteobacteria;c_Gammaproteobacteria;o_ Alteromonadales;f_Shewanellaceae												
Proteobacteria;c_Gammaproteobacteria;o_ Enterobacteriales;f_Enterobacteriaceae	y	y	y	y	y	y	y	y	y	y	y	y
Proteobacteria;c_Gammaproteobacteria;o_ Pseudomonadales;f_Moraxellaceae	y	y		y	y	y	y	y	y	y	y	y
Proteobacteria;c_Gammaproteobacteria;o_ Pseudomonadales;f_Pseudomonadaceae	y	y	y	y	y	y	y	y	y	y	y	y

Unique Famalies
Shared families in samples that were strong O157:H7 protectors
Shared families in samples that were weak O157:H7 protectors

Supplementary Figure 1

Examples of drain types samples collected from.

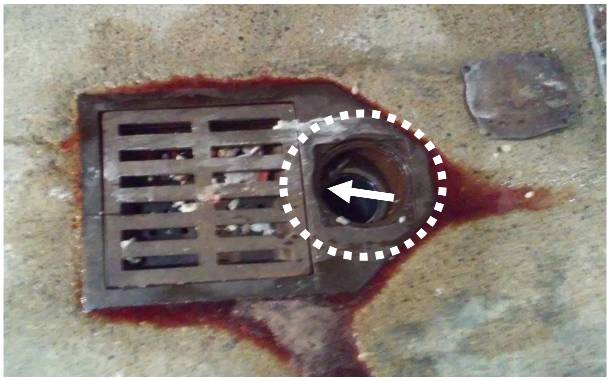

A. Side trap drain with cleanout cover removed (A10, A11).

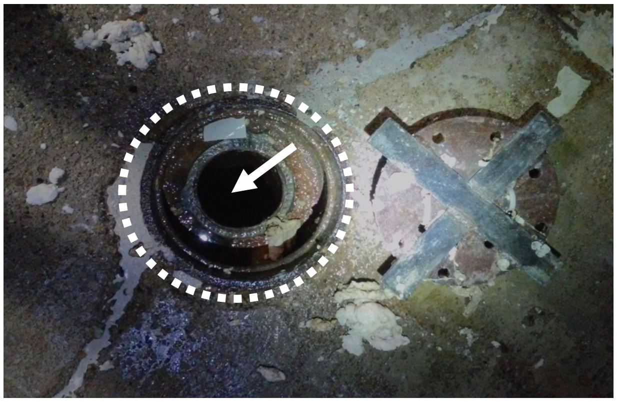

B. Standard industrial drain with top grate removed (A8, A9).

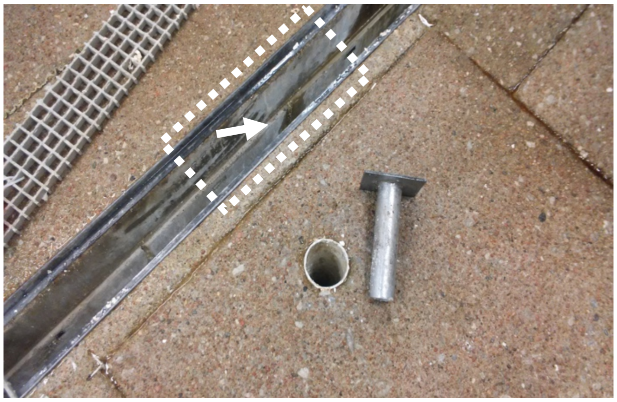

C. Trench drain with grate removed and cleanout opened (B8, B9, B10, B11).

Areas sampled are indicated by dashed line, with arrow indicating interior surfaces targeted for biofilm collection. Hotbox drain samples from Plant A were collected from side trap drains, while cooler drain samples were collected from standard drains. All drain samples from Plant B were collected from trench drains.
